# Development of an Injectable Slow-Release Metformin Formulation and Evaluation of Its Potential Antitumor Effects

**DOI:** 10.1038/s41598-018-22054-w

**Published:** 2018-03-02

**Authors:** Sara Baldassari, Agnese Solari, Guendalina Zuccari, Giuliana Drava, Sara Pastorino, Carmen Fucile, Valeria Marini, Antonio Daga, Alessandra Pattarozzi, Alessandra Ratto, Angelo Ferrari, Francesca Mattioli, Federica Barbieri, Gabriele Caviglioli, Tullio Florio

**Affiliations:** 1 Department of Pharmacy (DIFAR), University of Genova, 16148 Genova, Italy; 2 Department of Internal Medicine (DiMI), University of Genova, 16132 Genova, Italy; 3 IRCCS-AOU San Martino-IST, 16132 Genova, Italy; 4 Istituto Zooprofilattico Sperimentale del Piemonte, Liguria e Valle D’Aosta, National Reference Center of Veterinary and Comparative Oncology (CEROVEC), 16129 Genova, Italy; 5 Centre of Excellence for Biomedical Research (CEBR), University of Genova, 16132 Genova, Italy

## Abstract

Metformin is an antidiabetic drug which possesses antiproliferative activity in cancer cells when administered at high doses, due to its unfavorable pharmacokinetics. The aim of this work was to develop a pharmacological tool for the release of metformin in proximity of the tumor, allowing high local concentrations, and to demonstrate the *in vivo* antitumor efficacy after a prolonged metformin exposition. A 1.2% w/w metformin thermoresponsive parenteral formulation based on poloxamers P407 and P124, injectable at room temperature and undergoing a sol-gel transition at body temperature, has been developed and optimized for rheological, thermal and release control properties; the formulation is easily scalable, and proved to be stable during a 1-month storage at 5 °C. Using NOD/SCID mice pseudo-orthotopically grafted with MDA-MB-231/luc^+^ human breast cancer cells, we report that multiple administrations of 100 mg of the optimized metformin formulation close to the tumor site cause tissue accumulation of the drug at levels significantly higher than those observed in plasma, and enough to exert antiproliferative and pro-apoptotic activities. Our results demonstrate that this formulation is endowed with good stability, tolerability, thermal and rheological properties, representing a novel tool to be pursued in further investigations for adjuvant cancer treatment.

## Introduction

Metformin is the first-line treatment for type-2 diabetes^1^, which recent epidemiological evidence identified as potential, although still controversial, anti-tumor agent^2–4^. Metformin received increasing attention due to its potential antiproliferative properties since Evans *et al*.^5^ showed decreased cancer incidence in individuals with type-2 diabetes taking metformin, compared with patients under sulfonylureas or exogenous insulin treatments, which, on the other hand, also showed increased cancer-related mortality^6^. This anti-tumor effect was confirmed in preclinical studies, proposing that metformin might be used as adjunct to conventional cancer therapies in most human tumors to potentiate cytotoxic effects and overcome drug resistance^7–9^; thus the potential repositioning of metformin as anticancer drug is now object of several clinical studies^2,10^. Mechanistically, metformin antitumor effects are based upon a dual action, involving the correction of systemic hyperinsulinemia^11^ and the direct inhibition of cancer cell proliferation and viability, showing a significantly higher efficacy towards cancer stem cell subpopulations^12–17^. Pleiotropic inhibitory effects of metformin are mediated by multiple pathways in cancer cells^4,18^: metformin mainly acts through the activation of 5′ adenosine monophosphate (AMP)-activated protein kinase (AMPK) and the inhibition of the mTOR signaling pathway; moreover, it may cause direct inhibition of mitochondrial respiratory complex I, reducing ATP production, and induce antiangiogenic activity^19^. Recent studies highlighted that more cancer-specific molecular targets are also affected by metformin with high specificity, including the inhibition of the intracellular chloride channel 1 (CLIC1) activity^20,21^ and the dysregulation of proliferation-related miRNAs^22^. Furthermore, metformin treatment decreases the number and dimension of metastases^23^ specifically targeting cancer stem cells without toxic effects on normal cells, as shown in human breast epithelial cells^14,24^ or mesenchymal stem cells^16,20^. In fact, in an oncological context, one of the most relevant features of metformin is the satisfactory safety profile, being lactic acidosis its most serious, though very rare, side effect; conversely, hypoglycemia is not commonly observed either in type-2 diabetes patients or normal subjects^25^. Unfortunately, metformin is a class III drug according to The Biopharmaceutics Classification System (BCS), showing a quite unfavorable pharmacokinetic profile, having low and variable oral bioavailability (F = 55 ± 16%) and t_1/2_ of about 5 h^26^. Besides, *in vitro* studies showed that metformin inhibits cancer cell proliferation at concentration at least 10-fold higher than peak plasma concentration attained with typical dosing in diabetics^27^. Therefore, large doses would need to be orally administered to obtain anticancer effects with the consequent risk of either adverse effects or possible drug interactions in patients receiving chemotherapy. Based on these observations, a specific local delivery system could be useful to concentrate the drug at the tumor sites. Local administration may be achieved through systemic delivery of nanodispersed systems (liposomes, nanospheres, nanocapsules) which, however, have the drawbacks of low drug loading or potential sequestration by the reticuloendothelial system^28^; a viscous system, instead, could localize the release of the active agent close to the lesion, favoring the absorption of the drug by neoplastic cells. In the last decade increasing interest has been gained by water-soluble polymers able to form gels in the site of injection: these *in situ* gelling polymers are formulated as solutions, but at body temperature instantaneously form a strong gel capable of prolonging the residence time of the form releasing the active molecule. Thermally-induced gelling systems are attractive since they do not require organic solvents, copolymerization agents or externally applied gelation triggers under physiological conditions. Among the thermosensitive polymers, poloxamers, a series of triblock copolymers of ethylene oxide and propylene oxide, elicited wide interest for drug formulation; in particular, poloxamer P407, at appropriate concentrations in aqueous media, forms thermoreversible gels useful in several biomedical applications^29–32^.

Aimed to potentiate the efficacy of metformin for cancer treatment, here we report the development of different sterile metformin-loaded formulations based on poloxamers P407 and P124, which are injectable at room temperature (r.t.) and jellify at body temperature; these formulations were prepared according to statistical Design of Experiments (DoE) and characterized for thermal, rheological and drug release properties, in order to find an optimal formulation. We also measured the absorption kinetics of the optimized metformin preparation, evaluating plasma and liver concentrations of the drug after subcutaneous (s.c.) administration, and investigated its antitumor efficacy after peritumoral inoculation on mouse pseudo-orthotopic human breast cancer cell xenografts.

The relevance of this new pharmacological tool resides in providing the proof of the *in vivo* metformin anticancer efficacy after continuous local exposition to low drug concentrations. The developed local dosage form may be therapeutically applied for releasing, close to the lesion site, the minimum amount of metformin able to induce antitumor effects. Finally, the results of this study may be useful in the design of a long-releasing device for disease stabilization in inoperable cancer patients.

## Results and Discussion

### Development of thermoresponsive gel formulations

**Figure 1:**
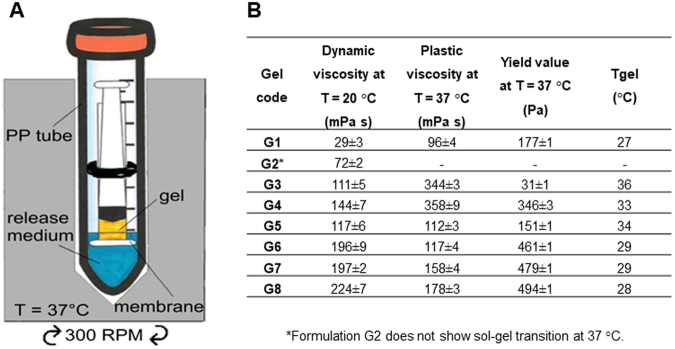
(**A**) Home-made device, based on an inverse syringe, for drug release study from an injectable gel. (**B**) Rheological properties of formulations G1-G8.

In an initial screening, parenteral forms known to localize and control drug release were investigated (i.e. albumin microspheres, inverse micelles, solid lipid nanoparticles), but none of them was able to load an adequate amount of metformin or control its release rate (data not shown), probably due to the high hydrophilicity of this small molecule. For this reason, our research focused on a biocompatible gel, possibly able to protract its residence time in the injection site and control metformin release through its highly viscous network. For the sake of easy and acceptable administration, the best dosage form appeared to be a thermosensitive system for s.c. administration using pre-filled syringes, fluid at r.t., but quickly turning into a compact gel at body temperature. Poloxamers, the most used thermoreversible systems in the pharmaceutical field, are ABA-type triblock copolymers composed of PEO (A) and PPO units (B) with molecular weights ranging from 1100 to 14,000 and PEO-PPO weight ratios varying from 1:9 to 8:2. Among poloxamers, P407 has been extensively used in drug delivery because its sol-gel transition occurs at relatively low concentration (16% w/w in pure water, at 27 °C)^33^ and it is well tolerated *in vivo*^34,35^. Since the gelation process of poloxamers can be influenced by the presence of other substances or additives^36^, the mutual interference between metformin solubility and gelation temperature of P407 formulation has been evaluated. The maximum dissolvable concentration of metformin, 17% w/w, proved not to inhibit the thermoresponsive gelation process of a 14% w/w P407 colloidal dispersion, which might permit a mild reduction of the minimum amount of copolymer necessary to have a suitable sol-gel transition at body temperature (16% w/w). Since this formulation has been conceived for a multiple dosage regimen of 100 mg/die/mouse, metformin has been dosed from 0.3 to 1.2% w/w, corresponding to approx. 1/15 and 1/4 of the mouse s.c. LD_50_ (225 mg/kg)^37^, respectively. We also measured the rate of drug release from the gels. Since release profile of semisolids is strongly influenced by the mechanical shear acting on the gel/liquid surface and by the volume of the medium used, a handy and cheap home-made device for studying drug release has been developed (Fig. 1A); it can be considered a bio-relevant system in terms of medium volume and shear rate applied. A gentle shaking of the medium, produced by orbital mixing, acting on the cellulose acetate porous membrane and not directly on the formulation, avoids any anomalous release caused by mechanical erosion. Moreover, the use of a chilling/heating dry bath, based on Peltier technology, allows an accurate temperature control (±0.2 °C), particularly important during the characterization of thermoreversible formulations. This method does not require replacement of withdrawn samples with fresh medium and is quite flexible in terms of the volume to be used (from 3 to 35 mL), guaranteeing sink conditions during the drug release while using the smallest volume possible. The first formulation considered, based on 16% w/w concentration of P407 loaded with 0.3% w/w of metformin (G1 –Table S1), showed a newtonian behavior at 20 °C and formed a gel with very low yield value at 37 °C, inadequately controlling the drug release (Fig. 1B). Indeed, this gel property should be as high as possible in the injected form to sustain the shear stress induced by body movements. To obtain a stronger gel, P188, a poloxamer widely applied in parenteral formulations, was added (G2), but the sol-gel transition of G2 did not occur at body temperature or lower. Other two P407/P188 formulations were prepared (G3-G4), increasing total poloxamer content: both showed low strength and thermal transition temperatures too close to the body temperature, considering that the injected sol might not immediately reach the gelation temperature in the whole mass, producing a burst effect. To replace P188 with another poloxamer, with the aim of reducing the sol-gel temperature and increasing gel compactness, thus prolonging drug release, it is necessary to consider (i) the gelation mechanism and (ii) the dependence of the process on the rate of the heat transfer to the mass. The gelation process involves the formation of polymeric micelles, at the corresponding critical micellar temperature, as a result of the PPO block dehydration^34^, and subsequent gel formation as the micelle concentration approaches a micellar close-packed organization characterized by a critical volume fraction of at least 0.53. The gelation temperature depends also on the PPO/PEO units ratio and decreases with increasing molecular weight of the PPO moiety contained in the copolymer^38^. Thus, 16% w/w P407 and P188 solutions were analyzed by photon correlation spectroscopy (PCS). In the P407 solution, aggregated structures, possibly corresponding to micelles (mean hydrodynamic radius = 0.7 nm; polydispersity index, PI = 0.62), were detectable, while in the P188 solution structures with higher mean hydrodynamic radius (50–150 nm, and PI = 0.9) were found, likely indicating a multiple association process. As a work hypothesis, it was considered that poloxamers having PPO/PEO ratio higher than P188 would form micelles with hydrodynamic radius comparable to the micelles formed by P407, which could increase gelation temperature and gel stability as compared to a gel containing only P407. Indeed, gel strength depends on close micelle packing and large chain entanglements, which prevents the separation of micelles from one another^39^. Therefore, poloxamer P124 was selected for further evaluation, since its 16% w/w solution has a mean micellar size (0.5 nm, PI = 0.52) similar to P407. The size of P124 micelles is due to a large PPO domain (53%^40^) and small MW with narrow distribution. Importantly, a solution containing equimolar amounts of P124 and P407 showed mixed micelles with average hydrodynamic radius of approx. 4 nm (PI = 0.58), whereas a solution containing equimolar amounts of P407 and P188 showed micelles with higher mean hydrodynamic radius (8.6 nm, PI = 0.53), which justifies the high gelation temperatures and low strength of these gels. The replacement of P188 (G2) with P124 (G5) made the sol-gel transition evident at body temperature (34 °C), although the resulting gel was weak. Comparing formulations containing higher amount of poloxamers, namely G4 *vs*. G6, a reduction of gelation temperature from 33 to 29 °C and the increase of yield value from 346 to 461 Pa were observed for the P124 formulation. Probably, the presence of the small P124 molecules enlarges the average size of P407-P124 mixed micelles, increasing the fraction volume of solution occupied by organized structures, while the presence of the long PEO chains in P407 molecules strengthens the micelle shell entanglement. G6 released approximately 60% of loaded metformin in 6 h, and the release profile appeared improved if compared to G1 (Fig. S1). Therefore, on the basis of these promising properties and of the relatively low poloxamer content, G6 was used as reference formulation for further modifications. With the aim of testing the formulation in mice, metformin content was increased up to 1.2% (G7 and G8) to maximize the effectiveness of multiple 100 mg gel administrations. Both formulations did not show a release profile significantly different from G6, suggesting that the mechanism controlling drug release rate is not influenced by the drug load within the evaluated range. Instead, the rheological properties, especially yield value and plastic viscosity, appeared to depend on metformin content, which might justify the decrease of gelation temperature for the 1.2% metformin content (G8) (Fig. 1B).

### Properties of the thermosensitive formulations and their optimization

**Figure 2:**
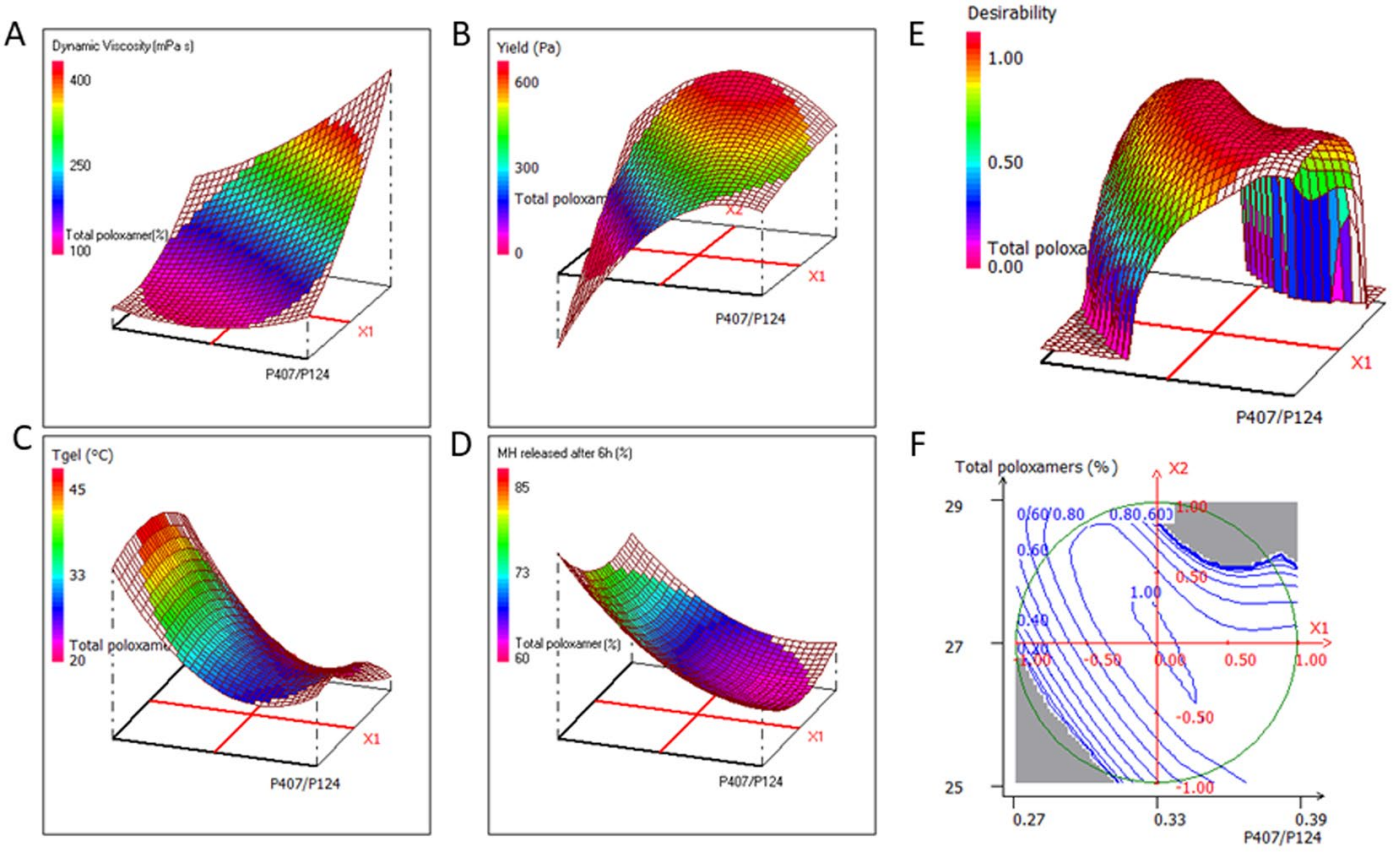
Response surfaces for: (**A**) dynamic viscosity at 20 °C; (**B**) yield value at 37 °C; (**C**) gelation temperature; and (**D**) % of metformin (MH) released after 6 h. Desirability surface and corresponding contour plot are reported in (**E**) and (**F**).

To develop the best formulation in terms of prolonged release of metformin, we investigated the experimental domain around G8, studying the effect of total amount of poloxamer and P407/P124 molar ratio on the injectable dosage form. A Doehlert experimental design^41^ was applied setting the amount of P407 in the tested formulations between 15% and 20%, a range supposed to allow the sol-gel transition at physiological temperatures (Table S2). The chosen limits for P407/P124 molar ratio were from 1:3.7 to 1:2.6. Seven formulations were tested; for each formulation five responses, describing rheological and thermal properties (dynamic viscosity at 20 °C, plastic viscosity at 37 °C, yield value at 37 °C, gelation temperature and rate), and one related to release control (% of metformin released after 6 h), were measured. The release profiles of some of the DoE formulations are shown in Fig. S2. The mathematical model describing the relationship between the two formulation parameters and the gel properties allowed to obtain the response surfaces, four of which are shown in Fig. 2A–D. Low dynamic viscosity at 20 °C (Y1) is required to freely inject the formulation, assuming that the refrigerated formulation is administered only after being conditioned at r.t. This response increases with P407/P124 molar ratio and with total poloxamer amount (Fig. 2A), being P407 characterized by higher molecular weight, thus influencing formulation compactness more than P124. High plastic viscosity at 37 °C (Y2) allows to reduce the possibility of a rapid and uncontrolled diffusion of the drug when the formulation is stressed over its yield value at the injection site. Plastic viscosity increases with P407/P124 molar ratio and, to a lesser extent, with the amount of poloxamers, however it is preferable to avoid too high amounts of polymers, in particular P407, being responsible for high dynamic viscosity at r.t. Yield value at 37 °C (Y3) should be as high as possible to maximize the resistance to mechanical stress when the dosage form has been injected into the tissue. This response increases with P407/P124 molar ratio and/or total poloxamer content; however, the formulations corresponding to the central region of the experimental domain show high values of yield avoiding the use of high amounts of polymers (Fig. 2B). Tgel (Y4) is the minimum temperature at which the sol-gel transition is observed. This value should be high enough to allow easy handling of the formulation before administration; nevertheless, a too high value *in vivo* could determine a delay in gelation, with consequent immediate release of the drug from the injected sol. Optimal values of this property might be only theoretically close to 37 °C, but, for the above-mentioned reason, a temperature between 27 and 33 °C could assure a rapid gel formation (Fig. 2C). The amount of metformin released after 6 h (Y5) decreases when P407/P124 ratio increases, especially at low poloxamer ratio values, whereas the formulations at mean and high poloxamer ratios have similar drug release rates (Fig. 2D), as observed comparing the performance of G8 and G12 (64 *vs*. 60%). The gelation rate (Y6) depends on heat transfer rate through the polymer solution and is maximum at high P407/P124 ratio, and total poloxamer content. Formulations with low values for both parameters do not form gel (G9). Considering the advantage of limiting the amount of polymers also for this response, the optimal formulation is at the center of the experimental domain. Starting from these results, the choice of the best formulation was mainly based on the rheological features, preferably keeping the poloxamer content as low as possible, also considering the multiple administration regime to be applied. To simultaneously optimize the different gel properties, a multi-response approach was adopted by overlaying the contour plots, as shown in Fig. S3 for some relevant responses. Since overlaying more than two contour plots results in graphs of difficult interpretation, the multi-criteria approach, based on desirability function^42^, was applied: each response is transformed into a 0-to-1 scale, where 0 is totally undesirable and 1 is the optimal response, and the overall desirability is obtained combining the desirability functions of each response. Considering as optimal the formulations showing dynamic viscosity at 20 °C lower than 200 mPa s, plastic viscosity at 37 °C higher than 100 mPa s, yield value at 37 °C higher than 350 Pa, gelation temperature between 27 and 33 °C, metformin released after 6 h less than 65% and gelation rate higher than 25 mPa, the maximum desirability area (Fig. 2E,F) corresponds to the center of the experimental domain, thus G8 formulation was chosen for the *in vitro/in vivo* evaluation, since it falls within the maximum desirability area and it has a relatively low amount of poloxamers.

### Stability studies

All gels were sterilized by filtration under laminar flow hood and the rheological and release control properties before and after sterilization were compared: both titer and rheological parameters were not affected by the sterilization process. During the stability study carried out on a G8 batch stored in 1-mL syringes for 1 month at 5 °C, no modifications in gel appearance were detected, the titer did not change significantly, and no metformin degradation products were found in the chromatograms. In conclusion, the gel is adequately stable when stored in the fridge, for at least one month.

### *In vivo* biodistribution studies

**Figure 3:**
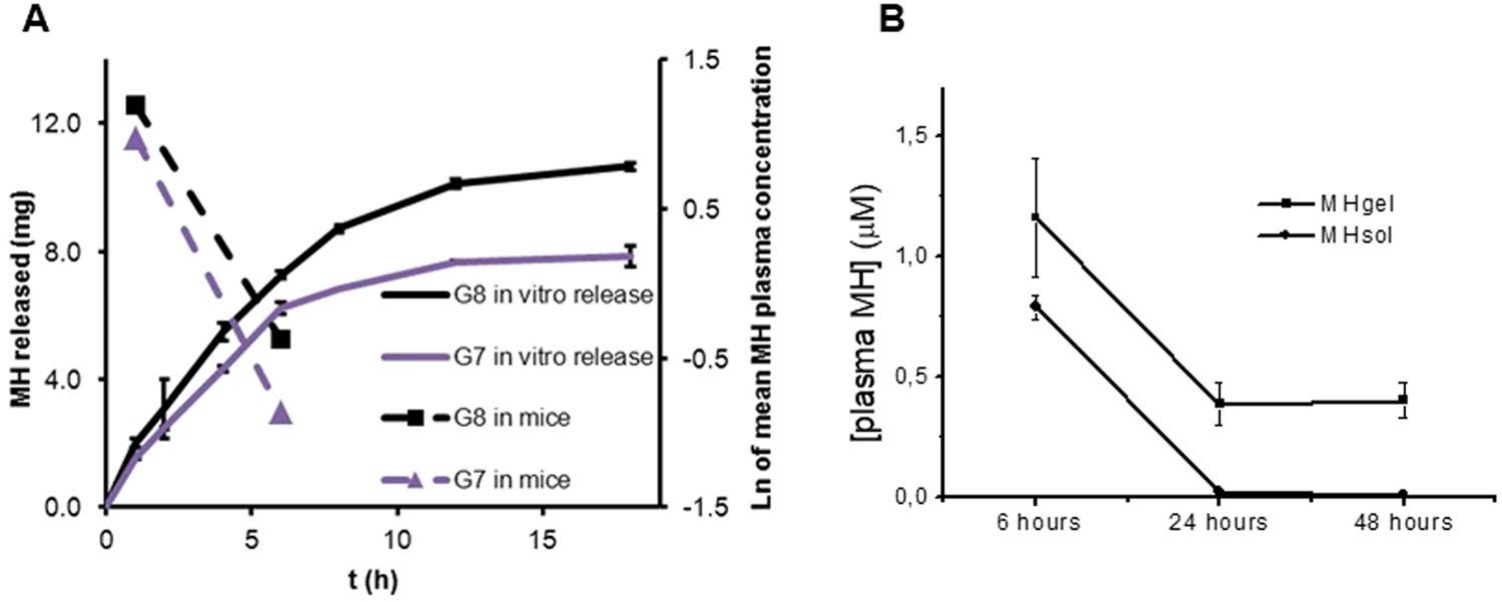
(**A**) Comparison of *in vitro* metformin hydrochloride (MH) release curves and mean plasma concentrations in mice 1 and 6 h after administration of G7 and G8 formulations. (**B**) Metformin (MH) plasma concentrations after single s.c. administration of 100 mg of gel-loaded formulation G8 (MH Gel), or water-soluble preparation (MH Sol) at the same concentration. Plasma levels were determined after 6, 24, and 48 h.

After multiple dosage administration regimen (100 mg/day for two weeks), mice showed good health, as only local irritation at the injection site was observed, that did not cause behavioral alterations, proving that the formulations were relatively well tolerated. Metformin plasma concentrations, 1 and 6 hours after the last administration, were 2.65 ± 0.4 and 0.42 ± 0.2 µg/mL for G7; 3.31 ± 0.3 and 0.69 ± 0.5 µg/mL for G8 (Fig. 3A). Considering a steady state distribution volume of metformin in mice of 1840 mL/kg^43^, the mean percentage release at 1 and 6 h is shown in Table S3. The percentage of metformin released after 1 h *in vivo* was comparable with that obtained *in vitro*, proving the reliability of the in-house built release device. To verify the ability of the metformin gel formulation to grant long-lasting drug concentrations, we measured plasma metformin concentrations 6, 24, and 48 h after a single s.c. injection. After 6 h, high concentrations were observed in mice plasma and, interestingly, lasted at significant levels up to 48 h (Fig. 3B). Metformin aqueous solution was s.c. injected at the same concentration as control. The concentrations measured 24 and 48 h after s.c. injection were much lower than those measured with metformin gel formulation, resulting constantly under the limit of quantification (LOQ < 0.1 µM) (Fig. 3B). This result indicates that a prolonged release of the drug is likely obtainable after metformin gel administration, increasing the persistence of the drug in the body.

### Evaluation of the antitumor activity of metformin thermosensitive gel

**Figure 4:**
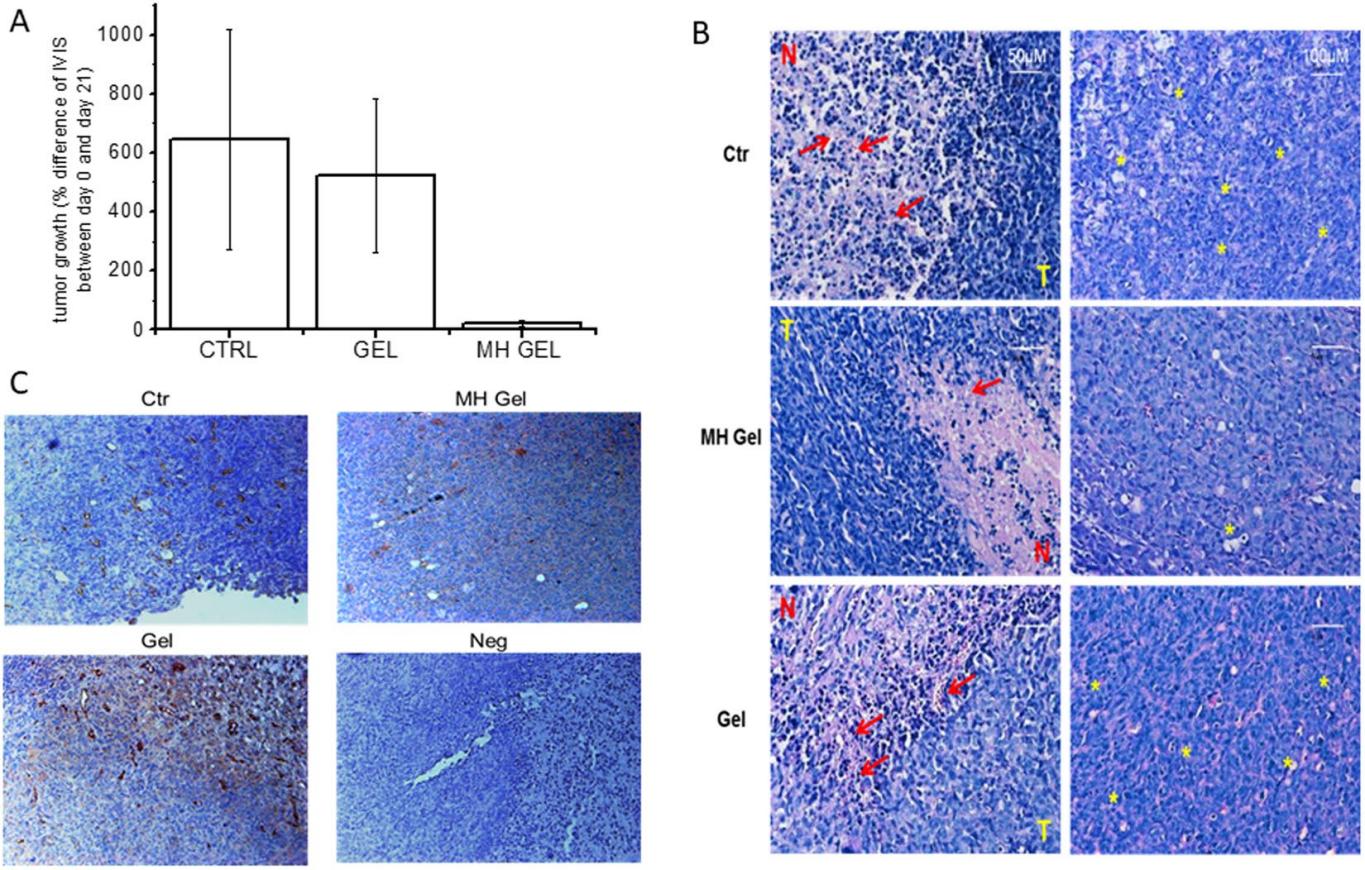
Antitumor activity of metformin gel formulation. (**A**) Difference of the luciferase emission (IVIS) before (day 0) and at the end of the treatment (day 21) from untreated mice (CTR), treated with placebo (Gel), or metformin-loaded gel (MH Gel). (**B**) Histological analysis of representative MDA MB231 tumors developed in control mice (CTR, untreated), in mice treated with metformin-loaded gel (MH Gel) or placebo (Gel). Images are representative of sections from different tumor areas, showing similar results. T = tumor area; N = necrotic area; red arrows = hemorrhagic areas and picnotic nuclei; yellow asterisks = representative mitotic cells. Bars = 50 µm. (**C**) Immunohistochemical analysis of representative MDA MB231 tumors developed in control mice (Ctr, untreated), in mice treated with metformin (MH)-loaded gel or placebo, stained with anti-CD31 antibody to highlight tumor vessels. Negative control (Neg) section was stained with the secondary antibody only. Bars = 100 µm.

To test possible applications of this new metformin formulation as anticancer treatment, the gel was administered to mice in which breast tumors had developed after pseudo-orthotopic injection of the human breast adenocarcinoma cells MDA-MB-231^44^; to obtain a measurable evaluation of tumor growth at the beginning and at the end of the treatment, cells were transduced with a luciferase-expressing retrovirus (luc^+^) that allows *in vivo* monitoring of cell growth by IVIS bioluminescence assay. In a first pilot experiment, using small groups of animals (n = 3), five days after cell injection tumor masses were evaluated by IVIS, and mice randomly divided into 3 groups, receiving the metformin-containing gel (100 mg/injection), the placebo gel or nothing (the untreated sub-group served as control), respectively. The treatment was performed 12 times, on every other day. Due to the peculiar absorption characteristics of metformin, which requires specific transporters that are often overexpressed in tumors^45^, we injected the drug peritumorally to favor its accumulation within the tumor mass and maximize the antiproliferative activity. All mice survived till the end of the study, which was stopped when control animals started to show signs of suffering due to the excessive tumor extension (after 21 days). Mice were monitored for potential systemic toxicity by evaluation of body weight throughout the treatment time, and gross examination of the liver, spleen, lungs, heart and kidneys of treated mice was carried out at the end of the experiment. Besides a moderate local inflammatory response in site of injection of both metformin and placebo gels, no alterations in the weight and toxic effects affecting morphologic appearance of the main internal organs were observed, as compared to control group. The antitumor effect of the injectable metformin formulation was evaluated, before the sacrifice, by measuring tumor volume by IVIS. We observed a reduction in the chemiluminescence of the tumor treated with the metformin gel formulation as compared to control mice, but not when the treatment was performed with the placebo gel, although due to the small number of treated animals the differences were not statistically significant (Fig. 4A). We also analyzed the explanted MDA-MB-231 tumors after hematoxylin/eosin staining, to confirm the efficacy of the treatment on tumor growth. In particular, the tissue structure of untreated tumors was compact and homogeneous, with a large number of irregularly arranged tumor cells, and a high number of mitoses (Fig. 4B). Large areas at the center of the tumors were characterized by cell overgrowth, necrosis, and blood extravasation into the parenchyma, while high content of large vessels was identified at tumor periphery in highly proliferating zones (Fig. 4B). These latter results were also confirmed by CD31 immunostaining (Fig. 4C). The same features, typical of rapidly growing tumors, were also observed in tumors treated with the placebo gel (Fig. 4B,C). Conversely, metformin-treated tumors showed lower cell density, significantly reduced number of mitosis and peripheral vessels, and necrotic areas surrounded by cells bearing picnotic nuclei, likely representing apoptotic cells (Fig. 4B). Moreover, a significant reduction of CD31+ vessels was observed in metformin-treated tumors (Fig. 4C). All these data are indicative of the antitumor effect induced by metformin gel treatment.

**Figure 5:**
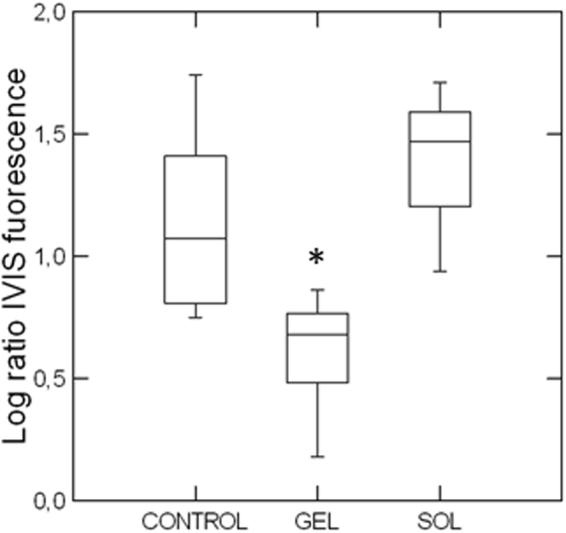
Box-and-whiskers plot showing the logarithmic transform of the ratio between the IVIS fluorescence at the end and at the beginning of the treatment in the three experimental groups: control, gel (treatment with metformin-containing gel 100 mg/ml) and sol (treatment with metformin-aqueous solution 100 mg/ml). Seven animals per group were s.c. injected with MDA-MB-231 cells that were allowed to grow till a visible tumor developed (7 days). Then, the volume of the tumors was evaluated by IVIS and the mice randomly divided in the three experimental groups, in a way that the chemiluminescence values were similar among groups. Then mice were treated every other day for 15 days with 100 mg of metformin formulations. At the end of the treatment, a second IVIS measurement was performed. The gel formulation caused a reduced tumor growth that was significantly different according to post-hoc tests (Tukey’s, Bonferroni, Fisher’s LSD, Scheffé, *p < 0.05) from both control and solution treatment. In each box, the central line marks the median of the data; the box edges indicate the first and third quartiles. The whiskers show the range of values falling within the inner fences (i.e. interquartile range multiplied by 1.5)^56^.

Subsequently, to perform a better statistical analysis and to compare the new formulation with the effects of metformin administered as aqueous solution, we performed a second series of experiments increasing the number of treated animals. In particular, three groups of seven MDA-MB-231 tumor-bearing mice were treated s.c. with metformin gel or aqueous solution (100 mg every 2 days), or left untreated (control group). At odds with the initial experiment, in this experiment the treatment had to be stopped after 15 days due to tumor overgrowth in control animals. Metformin antitumor effects were tested by IVIS. As shown in Fig. 5, comparing the log-transformed ratio between luciferase emission of each animal at the end and before the treatment, a significant reduction (*p* < 0.05) in tumor growth was observed in the animals treated with the metformin gel formulation, while the administration of the drug as aqueous solution did not interfere with tumor development, reaching an increase in volume comparable with that obtained in untreated animals (controls). At the moment of the treatment tumors were already developed in mice (diameter of about 1–1.5 mm), thus supporting the efficacy of the treatment for localized disease. However, due to the duration of the experiments, we do not have evidence that this approach could also be efficacious in metastatic disease and further studies will be required to verify this issue.

**Figure 6:**
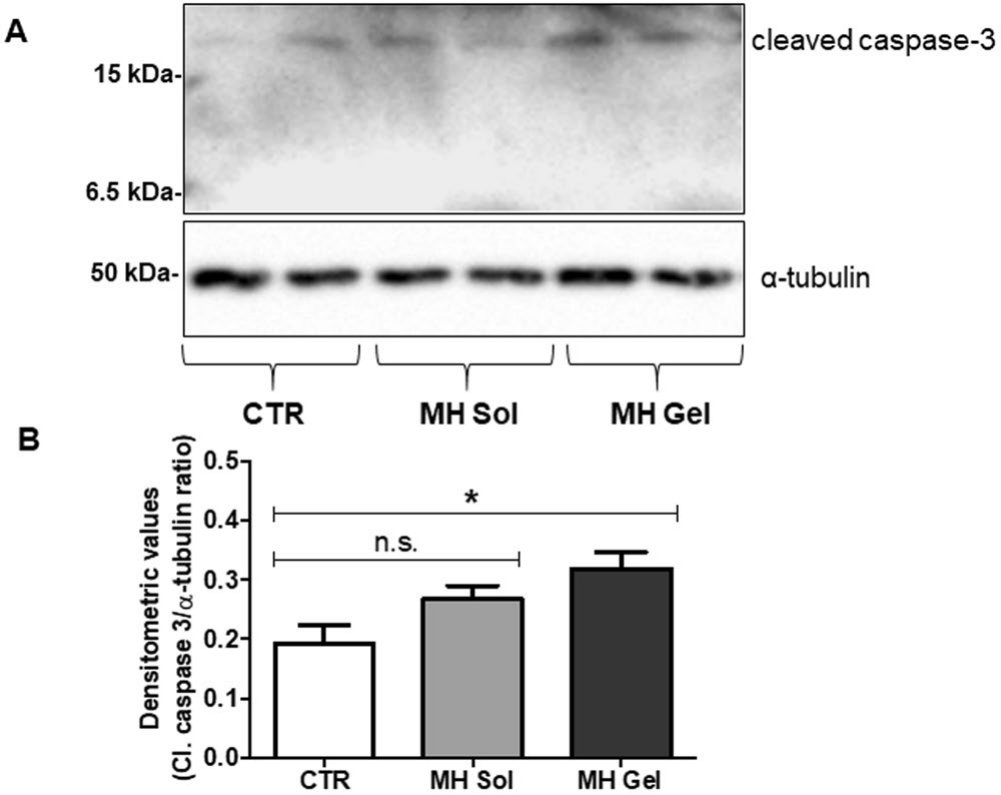
Metformin gel formulation induces apoptosis activation in mice tumors. (**A**) Representative Western blots of extracts from MDA-MB-231 tumors developed in untreated control mice (CTR), in mice treated with metformin aqueous solution (MH Sol) or metformin-loaded gel (MH Gel), using the antibody against the active fragment of caspase-3 (Cleaved caspase-3). Blots were stripped and reprobed with anti-α-tubulin antibody to normalize for differences in protein loading. (**B**) Densitometric analysis of Cleaved caspase-3 bands. Data presented as mean ± S.E.M from independently prepared tumor lysates (ANOVA with Dunnett’s post-hoc test: n.s. = not significant, *p < 0.05).

**Figure 7:**
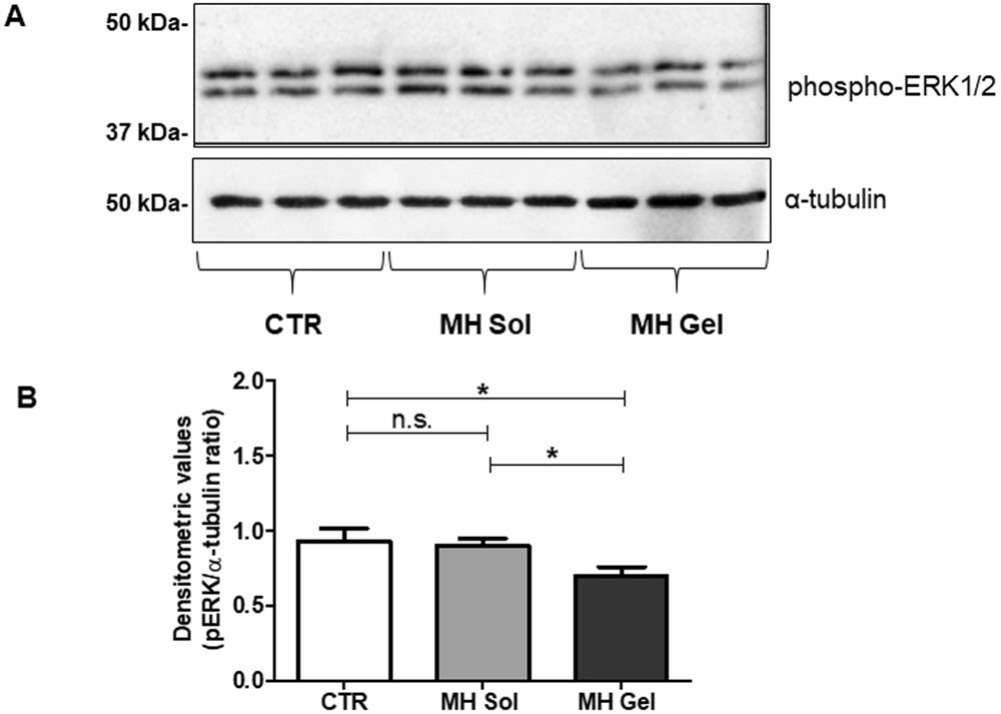
The metformin gel formulation modulates the ERK1/2 MAP kinase phosphorylation state in mice tumors. (**A**) Representative Western blots of extracts from MDA-MB-231 tumors developed in untreated control mice (CTR), in mice treated with metformin aqueous solution (MH Sol) or metformin-loaded gel (MH Gel), using the antibody against phospho-ERK1/2. Blots were stripped and reprobed with anti-α-tubulin antibody to normalize for differences in protein loading. (**B**) Densitometric analysis of Western blots performed in MDA-MB-231 tumors (n = 7). Values are reported as the means ± S.E.M. of densitometric measurement of the immunoreactive bands (ANOVA with Dunnett’s post-hoc test: n.s. = not significant, *p < 0.05).

Western blot analysis performed on metformin-treated tumors showed an increased expression of the active (cleaved) form of caspase-3 of about 68% (*p* < 0.05 *vs*. controls), while a lower (non-statistically significant) increase was observed after administration of metformin aqueous solution (average values after densitometric analysis of tumors derived from six animals, normalized for α-tubulin expression: controls 0.19 ± 0.06; metformin aqueous solution: 0.26 ± 0.05; metformin gel: 0.32 ± 0.07; representative gels and their quantification are reported in Fig. 6). From this analysis it is evident that s.c administration of metformin gel formulation, but not as aqueous solution, impairs tumor growth, likely through the induction of apoptosis, confirming the IVIS data. To further analyze the effect of the tissue concentrations reached after prolonged treatment, we evaluated the activation status of the MAP kinase ERK1/2 on explanted tumors from treated animals, since the antiproliferative effects of metformin might be associated to a reduced phosphorylation/activation of ERK1/2. As reported in Fig. 7, only metformin gel formulation induced a statistically significant inhibition of ERK1/2 phosphorylation, while when the drug was administered as aqueous solution no changes in ERK1/2 activation were observed. Importantly, samples treated with placebo (gel) showed the same levels of both caspase 3 and ERK1/2 activation as controls, further confirming the validity of the data here reported (Fig. S4).

**Figure 8:**
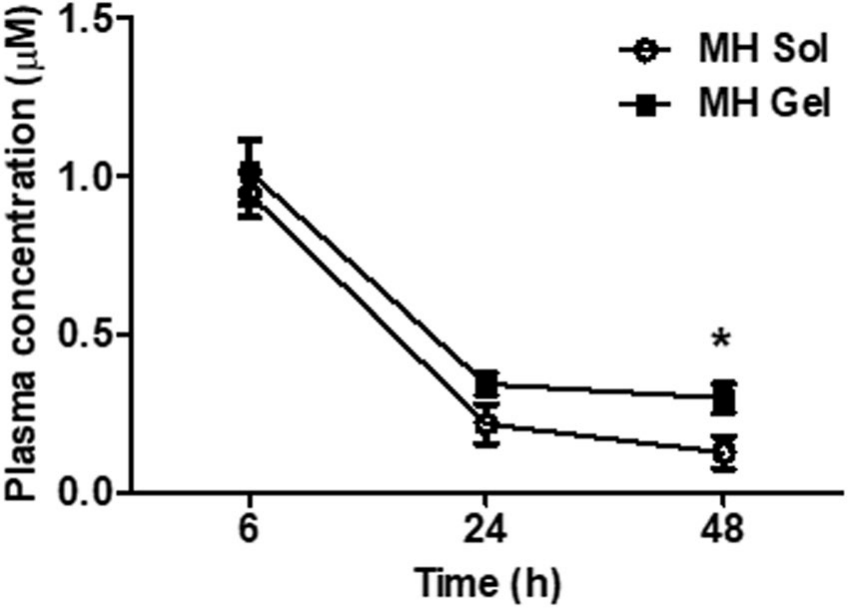
Metformin (MH) plasma concentrations after s.c. administration of 100 mg of metformin solution (MH Sol) or metformin-loaded gel (MH Gel) at the same concentration for 15 days. Values are reported as means ± S.E.M. of the measurement of plasma concentration from 7 mice (t-test: *p < 0.05 *vs*. MH Sol).

**Figure 9:**
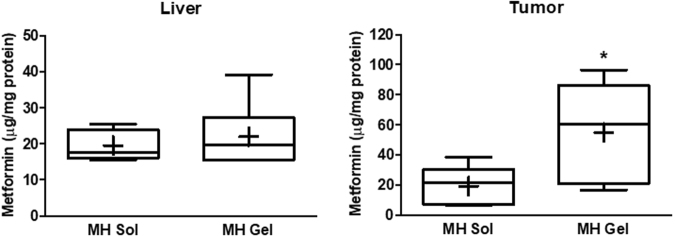
Metformin concentrations in liver and tumors measured in mice bearing MDA-MB-231 breast cancer xenografts following treatment with metformin aqueous solution (MH Sol) or metformin-loaded gel (MH Gel) injected s.c. peritumorally. Box-plots include the line at the median, boxes extend from the 25th to 75th percentiles, whiskers show range of data (min to max), a “+” indicates the mean. Results represent the data from individually treated mice (n = 7) (two-sample t-test: *p < 0.05).

To correlate the different efficacy of metformin solution and gel formulations with pharmacokinetic data, we compared metformin concentration in plasma after prolonged treatment. In particular, at the end of 15-day treatment, before the sacrifice, mice underwent blood sampling 6, 24 and 48 hours after the last injection. As reported in Fig. 8, repeated administration of the gel formulation showed similar kinetics as the single injection (see Figs 8 *vs*. 3B); conversely, a more prolonged persistence of metformin in plasma occurred after repeated *vs*. single injection of s.c. aqueous solution, being the former still detectable after 48 h. This result could be explained with the drug accumulation within red blood cells^46^ that was more consistent after repeated treatment. Moreover, we did not observe differences in metformin plasma levels after prolonged s.c. treatment with both formulations up to 24 h after the last injections. Conversely, metformin plasma concentrations 48 h after gel administration remained stable, while after aqueous solution injection they started to decrease, reaching a statistically significant difference versus the gel formulation (Fig. 8). However, although a sustained persistence in the bloodstream was obtained after administration as gel formulation, metformin plasma concentrations were not very high, and the difference with metformin solution was not so significant to justify the different clinical effect observed. Thus, we measured metformin liver and tumor concentrations, 48 h after the last injection, according to the reported tissue accumulation of the drug. In this analysis, a high accumulation of the drug within tissues was detected, and particularly in the tumor mass (Fig. 9). In particular, metformin liver concentrations, although clearly detectable in both treatments, were comparable between the two formulations, in agreement with the similar plasma levels. On the other hand, metformin concentration observed in tumors treated with gel formulation was 2.9-fold higher than that obtained with the aqueous solution (*p* < 0.05). This observation suggests that only the metformin gel formulation could lead to reach local (intratumor) concentrations high enough to exert antitumor effects. Moreover, the long-lasting tissue persistence of metformin is in agreement with previous studies demonstrating that metformin plasma levels are not a reliable index of the body content of the drug, and that metformin tissue accumulation can actually account for all its pharmacological effects (included the anti-hyperglycemic activity, which can be boosted by high concentrations of metformin in the liver^47^). Overall, these data provide several relevant information: 1) administration of metformin peritumorally using a slow-releasing drug delivery system may represent a valuable approach to use metformin as antitumor agent due to its ability to accumulate within tissues; 2) metformin plasma levels are less relevant for its pharmacological effects, suggesting that tissue accumulation may allow to obtain higher and long-lasting concentrations that are ultimately responsible for the pharmacological effects of the drug; 3) the reported discrepancies between the *in vitro* concentration required to induce antitumor effects and the obtainable plasma concentration *in vivo* may be reconciled by the consistent high tissue concentration of metformin we observed.

## Conclusions

A thermoresponsive gel formulation based on P407 and P124 poloxamers has been developed and optimized for release rate, thermal and injectable properties, proving to be suitable for multiple s.c. administration of metformin. This formulation can be manufactured in asepsis and/or sterilized by filtration, is easily scalable and producible in a ready-to-use prefilled syringe, and is stable for at least 1-month storage at 5 °C. The gel is effective in controlling metformin release with approximately zero-order kinetics *in vitro* and at least to determine acceptably high circulation levels *in vivo*. Pharmacokinetic data demonstrated that significant plasma concentrations of metformin are detectable up to 48 hours from the administration and, more importantly, that a significant accumulation within the tissues occurs at levels largely exceeding those detected in plasma. The continuous release of metformin at low amounts may exert a therapeutic activity allowing a significant accumulation of the drug within the tumors, where it induces antiproliferative and pro-apoptotic effects. Thus this approach may represent a valuable way for administering metformin as antitumor agent, delivering enough drug to reach effective concentrations within the neoplasia, avoiding extremely high plasma levels and consequent side effects.

In conclusion, we report for the first time the possibility to use a s.c. prolonging-release metformin formulation that, administered every 48 hours, grants high tissue levels of the drug able to induce antitumor effects.

## Methods

### Materials and chemicals

See supplementary information.

### Cells and animals

Human triple negative breast adenocarcinoma cell line MDA-MB-231 was grown in Dulbecco’s modified Eagle medium (Euroclone, Italy) supplemented with 10% fetal bovine serum (GIBCO, Italy), 100 U/mL penicillin/streptomycin and 2 mM L-glutamine (Euroclone, Italy). Cells were transduced with L-LUC-IN2, a retroviral vector coding for the firefly luciferase, to allow IVIS monitoring of tumor mass, as described^48^. Non-obese diabetic severe combined immunodeficient (NOD-SCID) mice (6–8 weeks old; Charles River, Italy) were used to evaluate pharmacokinetics and antitumor activity of the metformin formulations. Animals were handled in agreement with Italian regulations for the protection of animals used for scientific purposes and guidelines of the Ethical Committee for Animal Experimentation of the IRCCS-AOU San Martino-IST (Genova, Italy). All the following *in vivo* procedures were reviewed and approved by Review Board of the IRCCS San Martino-IST and by the Italian Ministry of Health (n°338, DLvo 116/92), and are compliant with EU Directive 2010/63/EU for animal experiments.

**Dosage form preparation as sol or in prefilled syringe**

**Thermal and rheological characterization**

**Dynamic light scattering assay**

See supplementary information.

### *In vitro* drug release studies

For the preparation of the home-made device (Fig. 1A), a 5-mL syringe barrel was cut perpendicularly to the circular section, to remove the Luer adapter, then the plunger was re-inserted backwards in the barrel to exploit the finger flange as support to attach the membrane. An accurately weighed amount of gel (1 g) was loaded in the sol form in the section comprised between the flange and the plunger, then the loaded syringe was heated in a ventilated oven at 37 °C for 10 min to promote the sol-gel transition. A cellulose acetate membrane with 0.45 µm pore size (Sartorius AG, Germany) was then glued on the flange, the plunger was moved to put the gel in contact with the membrane for removing residual air, and finally the syringe was suspended, by a PS annular diaphragm, in a 50 mL PP centrifuge tube containing 5 mL of PBS, so that the membrane was just below the liquid surface. The tube was closed by a plastic screw cap pierced to allow temperature control by a Pt-100 thermal probe. The dissolution medium was kept at a constant temperature of 37.0 ± 0.2 °C and under soft orbital shaking (300 rpm) in a Peltier chilling-heating dry bath (Torrey Pines Scientific Inc., USA). For each sampling time (1, 2, 4, 6, 8, 12, and 18 h) 3 different devices were used, so that each time point in the release curves is the mean of 3 values. Metformin concentration in the dissolution medium was measured spectrophotometrically (Hewlett Packard 8453, USA) at 233 nm and the released drug was expressed as percentage w/w of the drug content. The metformin content (% w/w) in the gel was determined by dissolving 50 mg of the gel in 25 mL of PBS and analyzing the resulting dispersion by HPLC.

### Optimization study

Two independent variables were studied, according to Doehlert experimental design: X1 = P407/P124 molar ratio at 5 levels (from 0.27 to 0.39) and X2 = total amount of poloxamers (P407 + P124) at 3 levels (from 25 to 29% w/w). These formulations (see Table S2 in Supplementary Information), forming the vertices of a regular hexagon in the X1-X2 plane plus a replicated center point, were prepared in asepsis and sterilized by filtration, then characterized for rheological and release control properties, by measuring the following responses: Y1 = dynamic viscosity at 20 °C (mPa s); Y2 = plastic viscosity at 37 °C (mPa s); Y3 = yield value at 37 °C (Pa); Y4 = Tgel (°C); Y5 = amount of drug released after 6 h (% w/w); Y6 = gelation rate (mPa).

### Formulation stability tests

An adequate number of 1 mL syringes containing formulation G8 was stored at 5 °C for one month. At 0, 2, and 4 weeks, 5 syringes were taken from the refrigerator and the content of each (100 ± 2 mg) was dispersed in 2 mL of mQ/MeOH 90:10, filtered through a 0.45 µm Minisart cellulose acetate filter (Sartorius AG, Germany) and assayed by HPLC (HP1090, Hewlett Packard, USA), as reported with modifications^49^.

### *In vivo* safety studies

In a first set of experiments, G7 and G8 (0.9 and 1.2% w/w metformin, respectively) were tested for tolerability on 13 mice. A repeated administration of 100 mg of G7 was set, because its dose corresponded to 1/5 of the metformin s.c. LD50 value in the mouse (225 mg/kg^37^). The same amount of G8 was also administered, corresponding to approximately 1/4 of the s.c. LD50 value in the mouse. 5 mice were daily treated with 100 mg of G7, 4 mice with 100 mg of G8 and 4 mice with 100 mg of placebo for 2 weeks; 1 and 6 hours after the last administration, 0.2 mL of blood were taken from each animal to evaluate metformin plasma concentrations (Fig. 3A and Table S3 in Supplementary Information) and, after sacrifice, organs were explanted for toxicity analysis.

### Pharmacokinetics and antitumor activity experiments

Eleven NOD/SCID mice in which 5 × 10^6^ viable MDA-MB 231/luc^+^ cells were pseudo-orthotopically inoculated in the mammary fat pad. After 5 days, when palpable tumors were detected, animals were analyzed by IVIS to quantify the tumor volume before treatment. The next day, mice were randomized into 3 groups: 4 mice were treated every other day with 100 mg of placebo (empty gel), 4 with 100 mg of G8 injected s.c. in proximity of the tumor and 3 were used as controls. At the end of the treatment, mice were euthanized and sections from three representative areas of each tumor were collected per each group and processed for histology studies. In a second set of experiments the treatment was repeated comparing the metformin gel formulation treatment with metformin aqueous solution and controls (n = 7 per group). After 15 days of treatment before the sacrifice, the animals were subjected to IVIS, to quantify tumor mass, and blood samples (0.2 mL) were taken from each animal 6, 24, and 48 h after the last treatment, for metformin plasma concentration measurements. After the sacrifice tumors and livers were explanted for biochemical analyses and metformin concentration measurement

#### Extraction procedure and plasma sample analysis

The extraction was performed by adding 15 µL of ranitidine (60 µM) as internal standard and 500 µL of acetonitrile to the tube containing 100 µL of plasma or 100 µL of homogenized tissue. After centrifugation (16,000 × *g* for 10 min), the supernatant was evaporated at 45 °C under a nitrogen stream and reconstituted with 200 µL of water. Metformin concentrations were determined by HPLC, as reported^50^, using the automated Ultimate 3000 HPLC system (ThermoFisher Scientific Inc., USA) equipped with the chromatography software Chromeleon 7.0 SR1 (Dionex Softron GmbH, Germany). Chromatographic determination was achieved using a reverse-phase column LiChrospher100 [250–4 RP-18e (5 µm)] and a guard column LiChrospher100 [4-4 RP-18e (5 µm)] (VWR-Merck KGaA, Germany), with acetonitrile-potassium dihydrogen phosphate buffer pH 3.5 (34:66 v/v) and 5 mM SDS as mobile phase. The elution was carried out at 0.7 mL/min, with detection wavelength set to 236 nm. Calibration curve was constructed using drug-free plasma and/or tissue homogenates spiked with metformin at 1–10 µM concentrations; the results analyzed by linear regression gave R^2^ = 0.999

#### Western blot analysis

The tissues were lysed in RIPA buffer and processed, as reported^51^. Proteins (20 µg), transferred to PVDF membrane (Bio-Rad Laboratories) and probed with primary antibodies (cleaved caspase-3, phospho-p42/44 MAPK ERK1/2, and α-tubulin from Cell Signaling), were incubated with secondary antibodies and immunocomplexes detected by Western Chemiluminescent HRP Substrate^52^, and densitometric analysis was performed using the Image Quant software Chemi-Doc system (all from Bio-Rad Laboratories)^53^.

#### Immunohistochemical analysis

The tumors were fixed with 4% paraformaldehyde overnight and embedded in paraffin. Tumor sections (4 µm) were subjected to antigen retrieval by heating in citrate buffer (pH 6), as reported^54^. Non-specific immunoreactivity was blocked with 10% normal goat serum (Sigma-Aldrich, Italy) and the primary antibody (anti-CD31, Ventana Inc., USA) applied overnight at 4 °C. IHC was performed using the EnVision Dual Link System-HRP (Dako, Italy). Sections were counter-stained with haematoxylin/eosin (Sigma-Aldrich). Negative controls were performed substituting the primary antibody with NGS (Sigma-Aldrich). Immunostaining was evaluated by light microscopy (Coolscope, Nikon, Japan) to determine the antigen expression and localization^55^.

### Statistics

NEMRODW software (LPRAI, France) was used for experimental design and for the related statistical analysis and graphs. Data were analyzed by two-sample t-test or one-way ANOVA and post-hoc tests, using Systat 13 and GraphPad Prism 5.02. Statistical significance was established at *p* < 0.05.

## Electronic supplementary material

Supplementary Information
