# Supplementary material for: Development of an Injectable Slow-Release Metformin Formulation and Evaluation of Its Potential Antitumor Effects
Source: Sci Rep. 2018 Mar 2;8:3929. doi: 10.1038/s41598-018-22054-w (PMC5834504; doi:10.1038/s41598-018-22054-w)
Supplement: Supplementary file 1 — Supplementary Information [file 41598_2018_22054_MOESM1_ESM.pdf]

SUPPLEMENTARY INFORMATION TO

Development of an Injectable Slow-Release Metformin Formulation and Evaluation of Its Potential Antitumour Effects

Sara Baldassari, Agnese Solari, Guendalina Zuccari, Giuliana Drava, Sara Pastorino, Carmen Fucile, Valeria Marini, Antonio Daga, Alessandra Pattarozzi, Alessandra Ratto, Angelo Ferrari, Francesca Mattioli, Federica Barbieri, Gabriele Caviglioli, and Tullio Florio

DETAILED MATERIALS AND METHODS

(for reference list see main text)

Chemicals. Polymers, i.e. polyoxyethylene–polyoxypropylene copolymers poloxamer P407 (MW 9840-14600; Lutrol F-127), P188 (MW 7680-9510; Lutrol F-68) and P124 (MW 2090-2360; Lutrol L-44), were kind gifts from BASF (Germany); metformin hydrochloride and all other chemicals (analytical grade) were from Sigma-Aldrich (Italy). PBS was prepared by dissolving 8.00 g of NaCl, 0.2 g of KCl, 1.44 g of $Na_2HPO_4·2H_2O$ and 0.24 g of KH_2PO_4 per 1 L of deionized water (pH 7.2).

Cells and animals. Human triple negative, breast adenocarcinoma cell line MDA-MB-231 was grown in Dulbecco's modified Eagle medium (Euroclone, Italy) supplemented with 10% fetal bovine serum (GIBCO, Italy), 100 U/mL penicillin/streptomycin and 2 mM L-glutamine (Euroclone, Italy). Cells, cultured at 37 °C in a humidified atmosphere of 5% CO_2 in air, were transduced as described[48] with L-LUC-IN2, a retroviral vector coding for the firefly luciferase, to allow IVIS monitor of tumor mass. Non-obese diabetic severe combined immunodeficient (NOD-SCID) mice (6-8 weeks old; Charles River, Italy) were used to evaluate pharmacokinetics and antitumor activity of metformin formulation. Animals, housed in pathogen-free conditions, were handled in agreement with Italian regulations for the protection of animals used for scientific purposes and guidelines of the Ethical Committee for Animal Experimentation of the IRCCS-AOU San Martino-IST (Genova, Italy). All the subsequently described *in vivo* procedures were reviewed and approved by Review Board of the IRCCS San Martino-IST and by the Italian Ministry of Health (n° 338, DLvo 116/92), and are compliant with EU Directive 2010/63/EU for animal experiments.

Dosage form preparation as sol or in prefilled syringe. Gels were prepared by the cold method: accurate amounts of poloxamer P407 (in the range 15.6-19.3 % w/w), P124 (8.0-11.0 % w/w) and/or P188 (8.0-10.7 % w/w) were added to a cold metformin solution (0.3-17 % w/w), and the formulation was kept at 5 °C under gentle magnetic stirring up to complete dispersion. The solutions (5 °C) were sterilized by filtration through a 0.2 μm sterile cellulose acetate filter (Minisart NML code 16534, Sartorius AG, Germany). The formulations studied are listed in Table S1.

Table S1. Composition of the different formulations studied (amounts expressed as % w/w).

Gel code	Metformin	P407	P124	P188
**G1**	0.3	16.0		
**G2**	0.3	16.0		8.0
**G3**	0.3	16.0		10.7
**G4**	0.3	17.5		9.7
**G5**	0.3	16.0	8.0	
**G6**	0.3	17.5	9.7	
**G7**	0.9	17.5	9.7	
**G8**	1.2	17.5	9.7	
**G9**	1.2	16.1	10.9	
**G10**	1.2	18.0	11.0	
**G11**	1.2	19.3	9.7	
**G12**	1.2	18.4	8.6	
**G13**	1.2	16.6	8.4	
**G14**	1.2	15.6	9.4	

For *in vivo* experiments and stability study, adequate volumes of the formulations (approx. 120 μL), in the sol form, were weighed into 1 mL single-use sterile hypodermic syringes (PIC 25 G x 5.8”, Artsana, Italy) so as to dispense 100±2 mg. The syringes were filled and re-packed in aseptic conditions under laminar fume hood.

Thermal and rheological characterization. For rheological characterization a concentric cylinder viscometer (Phisica Haake Thermo Fisher Scientific Inc., USA) equipped with a Z2 device was used. For the determinations of dynamic viscosity (20 °C), plastic viscosity (37 °C) and extrapolated yield value of the plastic gels (37 °C), the formulations, stored at 5 °C in a refrigerator, were loaded in the viscometer and thermally equilibrated at the test temperature (20 or 37 °C) for 30 min before the measurement, then were submitted to an increasing shear rate from 0 to 500 s^{-1} by a 100 s^{-1}/min gradient.

For the gelation rate study, the samples, poured in the viscometer cup, were conditioned in the refrigerator at 5 °C for 20 min, then the cup was mounted in the viscometer, warming the sample at 37 °C, under constant shear rate of 100 s^{-1}, upon reaching a constant viscosity. The gelation rate (mPa) was measured as the first derivative of dynamic viscosity with respect to heating time.
The gelation temperature (Tgel) was determined using a Peltier chilling-heating dry bath (Torrey Pines Scientific Inc., USA). Approx. 0.5 mL of the sols were loaded in 1.5 mL Safe Lock tubes (Eppendorf, Germany) and stored in the refrigerator before the measurement. The tubes were placed in the appropriate block of the dry bath set at 25±0.2 °C and 600 rpm shaking rate. Every 15 min the cones were taken out and placed horizontally to observe the state of the sample, then put back in the block and the temperature was increased by 1 °C. Tgel was determined as the temperature when the meniscus would no longer move upon.
The results are expressed as mean of three replicates ± standard deviation (SD).

Dynamic light scattering assay. Particle size (mean hydrodynamic radius,) and polydispersity index (PI) were determined by dynamic light scattering using a photon correlation spectroscopy (PCS) assembly (Zetasizer 3000 HS, Malvern Instruments, UK). Determinations were carried out at 20 °C, at a fixed angle of 90°. Results are reported as mean of six measurements ± SD.

***In vitro* drug release studies.** For the preparation of the home-made device (see Fig. 1A), a 5-mL syringe barrel was cut perpendicularly to the circular section, to remove the Luer adapter, then the plunger was re-inserted backwards in the barrel to exploit the finger flange as support to attach the membrane. An accurately weighed amount of gel (1 g) was loaded in the sol form in the section comprised between the flange and the plunger, then the loaded syringe was heated in a ventilated oven at 37 °C for 10 min to promote sol-gel transition. A cellulose acetate membrane with 0.45 µm pore size (code 11106-25-N, Sartorius AG, Germany) was then glued on the flange, the plunger was moved to put the gel in contact with the membrane for removing residual air, and finally the syringe was suspended, by a PS annular diaphragm, in a 50 mL PP centrifuge tube containing 5 mL of PBS, so that the membrane was just below the liquid surface. The tube was closed by a plastic screw cap pierced to allow temperature control by a Pt-100 thermal probe. The dissolution medium was kept at a constant temperature of 37.0±0.2 °C and under soft orbital shaking (300 rpm) in a Peltier chilling-heating dry bath (Torrey Pines Scientific Inc., USA).
For each sampling time (1, 2, 4, 6, 8, 12 and 18 h) 3 different devices were used, so that each time point in the release curves is the mean of 3 values. Metformin concentration in the dissolution medium was measured spectrophotometrically (Hewlett Packard 8453, USA) at 233 nm and the released drug was expressed as percentage (w/w) of drug content. Metformin content (% w/w) of the

gel was determined by dissolving 50.0 mg of the gel in 25.0 mL of PBS and analyzing the resulting dispersion by HPLC as described in “Formulation stability tests”.

Optimization study. Two independent variables were studied, according to Doehlert[40] experimental design: X1 = P407/P124 molar ratio at 5 levels (from 0.27 to 0.39) and X2 = total amount of poloxamers (P407+P124) at 3 levels (from 25 to 29 % w/w). These formulations (see Table S2), forming the vertices of a regular hexagon in the X1-X2 plane plus a replicated centre point, were prepared in asepsis and sterilized by filtration, then characterized for rheological and release control properties, by measuring the following responses: Y1 = dynamic viscosity at 20 °C (mPa s); Y2 = plastic viscosity at 37 °C (mPa s); Y3 = yield value at 37 °C (Pa); Y4 = Tgel (°C); Y5 = amount of drug released after 6 h (% w/w); Y6 = gelation rate (mPa).

Table S2. Experimental design formulations.

Gel code	X1	X2 (% w/w)
**G8**	0.33	27
**G9**	0.27	27
**G10**	0.30	29
**G11**	0.36	29
**G12**	0.39	27
**G13**	0.36	25
**G14**	0.30	25

Formulation stability tests. An adequate number of 1 mL syringes containing formulation G8 was stored at 5 °C for one month. At 0, 2 and 4 weeks, 5 syringes were taken from the refrigerator and the content of each (100 ± 2 mg) was dispersed in 2 mL of mQ/MeOH 90:10, filtered through a 0.45 µm Minisart cellulose acetate filter (code 17598-Q, Sartorius AG, Germany) and assayed by HPLC (HP1090, Hewlett Packard, USA). The method was developed through modifications of the protocol described by Bretnall and Clarke[49]: Column RP 18, X-Bridge C18 (Waters, Italy), 3.5 µm, 4.6 x 150 mm, equipped with Waters X-Bridge BEH C18 Sentry Guard Cartridge precolumn, 3.5 µm, 4.6 x 20 mm; elution performed by linear gradient: 100% phase A for 3 min, linear gradient for 4 min up to

10% phase B, isocratic for further 6 min; 7 min of equilibration time at 100% of A; phase A was 18.5% v/v of MeOH – 81.5% v/v of 0.2% w/w sodium heptanesulfonate + 0.2% v/v triethylamine aqueous solution, whose pH was adjusted to 3.0 using ortophosphoric acid; phase B was acetonitrile; flow 1 mL/min, injection 10 µL; absorption was measured at 200 (dimethylamine), 213 and 233 nm. Standard solutions of metformin and its related substances (A, D, and F, corresponding to 1-cyanoguanidine, 1,3,5-triazine-2,4,6-triamine (melamine) and dimethylamine – European Pharmacopoeia 9th ed.) in the mobile phase were also analyzed for retention time and LOD, and the following data were obtained: RRT 0.138, 0.193, 0.653, 1 and LOD 257, 0.9, 1.19, 1.19 ng for dimethylamine, 1-cyanoguanidine, melamine and metformin, respectively. The linearity of metformin areas in the chromatogram was verified in the range 67.5-100 % of gel titre.

***In vivo* safety studies.** In a first set of experiments, G7 and G8 (0.9 % and 1.2 % w/w metformin, respectively) were tested for tolerability on 10 mice. A repeated administration of 100 mg of G7 was set, because its dose corresponded to 1/5 of the metformin s.c. LD50 value in the mouse (225 mg/kg)[36]. The same amount of the G8 was also administered, corresponding to approximately 1/4 of the s.c. LD50 value in the mouse. Five mice were daily treated with 100 mg of G7, 4 mice with 100 mg of G8 and 4 mice with 100 mg of placebo for 2 weeks (total 10 administrations; no treatments at weekends); one and 6 hours after the last administration 0.2 mL of blood were taken from each animal to evaluate metformin plasma concentrations (the mean values are reported in Table S3) and, after sacrifice, organs were explanted for toxicity analysis.

Table S3. Percent of *in vivo* metformin release of G7 and G8 formulations, 1 and 6 h after administration.

	**Metformin released (%) ± SD**	
	**1 h**	**6 h**
**G7**	11.6 ± 0.8	1.7 ± 0.9
**G8**	10.1 ± 1.0	2.1 ± 1.4

Pharmacokinetics and antitumor activity experiments.

Animal treatments

A second study was performed on 11 NOD/SCID mice in which 5x10^6 viable MDA-MB 231/luc+ cells,

resuspended in Matrigel (BD Biosciences, Italy), were pseudo-orthotopically inoculated in the mammary fat pad; mice were daily inspected for tumor appearance by visual observation and palpation, and monitored for any discomfort. After 5 days, when in all the animals a palpable mass was detected, the animals were analyzed by IVIS to quantify the tumor volume before treatment. The next day, the mice were randomized into 3 groups: 4 mice were treated every other day with 100 mg of placebo, 4 with 100 mg of G8 injected s.c. in proximity of the tumor and 3 were used as controls; no treatments were performed in the weekends. The total number of treatments was 12, and blood samples (0.2 mL) were taken from each animal 6, 24 and 48 h after the first treatment for metformin plasma concentration measurements. Metformin aqueous solution was s.c. injected at the same concentration of the gel formulation as control. At the end of the treatment, the animals were euthanized and tumors and livers explanted and snap frozen in liquid nitrogen. Three representative tumor tissues were collected per each group and processed for histology and biochemical studies.

Extraction procedure and plasma sample analysis

Extraction was performed by adding 15 μL of ranitidine (60 μM), as internal standard, and 500 μL of acetonitrile to the tube containing 100 μL of plasma or 100 μL of tissue previously homogenized in 1 mL of distilled water. After centrifugation at 16,000 x *g* for 10 min, the clear supernatant was transferred and evaporated to dryness at 45 °C under a nitrogen stream and reconstituted with 200 μL of deionized water. Concentrations of metformin in plasma and tissues were determined by HPLC with a previously, slightly modified, validated method[50]. An automated Ultimate 3000 HPLC system (Thermo Fisher Scientific Inc., USA) was used for HPLC analyses. Chromatography software Chromeleon 7.0 SR1 (Dionex Softron GmbH, Germany) was used for data acquiring. The chromatography determination was achieved using a reverse-phase column LiChrospher®100 [250-4 RP-18e (5 μm)] and a guard column LiChrospher®100 [4-4 RP-18e (5 μm)] (VWR-Merck KGaA, Germany), with acetonitrile-potassium dihydrogen phosphate buffer pH 3.5 (34:66 v/v) and 5 mM SDS as mobile phase. The elution was carried out at a flow rate of 0.7 mL/min at room temperature, with detection wavelength set to 236 nm. Calibration point curve was constructed using drug-free plasma and/or tissue homogenates spiked with metformin concentrations from metformin aqueous solution, and the results analyzed by linear regression giving a determination coefficient of R^2 = 0.999.

Western blot analysis

Tissues were lysed in RIPA buffer and the "Complete" protease inhibitor mixture (Roche Applied Science, Burgess Hill, UK) for 10 min at 4 °C. Nuclei were removed by centrifugation (5,000 rpm at 4 °C, for 10 min), and total protein content measured using Bradford assay (Bio-Rad Laboratories,

Italy)[51]. Proteins (20 μg) were resuspended in Laemmli buffer (2% SDS, 62.5 mM Tris, pH 6.8, 0.01% bromophenol blue, 1.43 mM 2-mercaptoethanol, and 0.1% glycerol), size-fractionated by SDS/PAGE, transferred to PVDF membrane (Bio-Rad Laboratories) and probed with primary antibodies (anti-phospho-ERK1/2, -Mcl-1, -cleaved caspase-3 (Asp175), and -α-tubulin, all from Cell Signaling, USA). Probed membranes were then incubated with appropriate anti-mouse or anti-rabbit IgG-horseradish peroxide-conjugated secondary antibody; immunocomplex detection and densitometric analysis were performed using the Immobilon Western Chemiluminescent HRP Substrate and the Image Quant software Chemi-Doc system (all from Bio-Rad Laboratories)[52]. α-tubulin was used as an internal control for protein loading.

Immunohistochemical analysis

Tumors were obtained from the mice as aforementioned, fixed with 4% paraformaldehyde overnight and embedded in paraffin. Paraffin-embedded tissue sections (4 μm) were deparaffinized and stained with hematoxylin (Sigma-Aldrich, Italy) to determine the histological structure. As reported[53], deparaffinized/rehydrated sections were subjected to an antigen retrieval protocol by heating in citrate buffer (pH 6). Non-specific immunoreactivity was blocked with 10% normal goat serum (Sigma-Aldrich, Italy) and primary antibody (anti CD31, Ventana Inc., USA) applied overnight at 4 °C. IHC was performed using the EnVision™+ Dual Link System-HRP (Dako, Italy). Sections were counterstained with haematoxylin (Sigma-Aldrich, Italy). Negative controls were run in parallel, substituting the primary antibody with NGS in Tris-buffered saline (Sigma-Aldrich, Italy). Immunostaining was evaluated by light microscopy (Coolscope, Nikon, Japan) to determine the antigen expression and localization[54].

Statistics. NEMRODW® software (LPRAI, France) was used for experimental design and for the related statistical analysis and graphs. Data (after logarithmic transform, when necessary) were submitted to two-tailed t-test or one-way ANOVA. Several post-hoc tests (Tukey's, Bonferroni, Fisher's LSD, Scheffè, Dunnett's) were applied, using Systat 13 and GraphPad Prism 5.02. Statistical significance was established at $p<0.05$.

Supplementary Tables and Figures

Table S1. Composition of the different formulations studied (amounts expressed as % w/w).

Gel code	Metformin	P407	P124	P188
**G1**	0.3	16.0		
**G2**	0.3	16.0		8.0
**G3**	0.3	16.0		10.7
**G4**	0.3	17.5		9.7
**G5**	0.3	16.0	8.0	
**G6**	0.3	17.5	9.7	
**G7**	0.9	17.5	9.7	
**G8**	1.2	17.5	9.7	
**G9**	1.2	16.1	10.9	
**G10**	1.2	18.0	11.0	
**G11**	1.2	19.3	9.7	
**G12**	1.2	18.4	8.6	
**G13**	1.2	16.6	8.4	
**G14**	1.2	15.6	9.4	

Table S2. Experimental design formulations.

Gel code	X1	X2 (% w/w)
**G8**	0.33	27
**G9**	0.27	27
**G10**	0.30	29
**G11**	0.36	29
**G12**	0.39	27
**G13**	0.36	25
**G14**	0.30	25

Table S3. Percent of *in vivo* metformin release of G7 and G8 formulations, 1 and 6 h after administration.

	**Metformin released (%) ± SD**	
	**1 h**	**6 h**
**G7**	11.6 ± 0.8	1.7 ± 0.9
**G8**	10.1 ± 1.0	2.1 ± 1.4

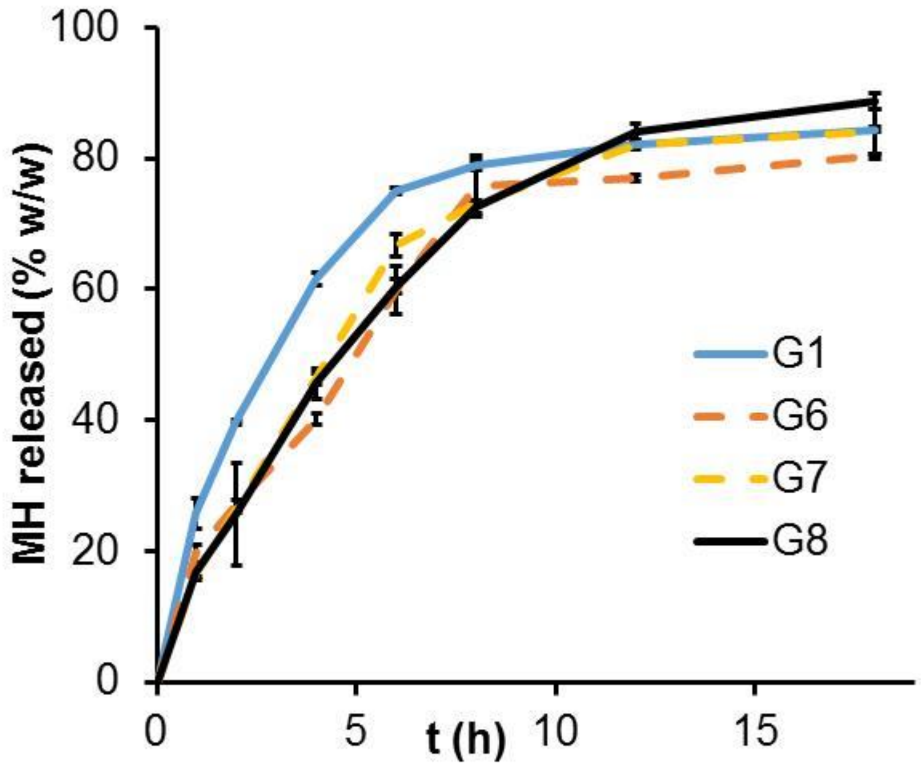

Figure S1. Metformin (MH) release curves of formulations G1, G6, G7, G8 (n=3).

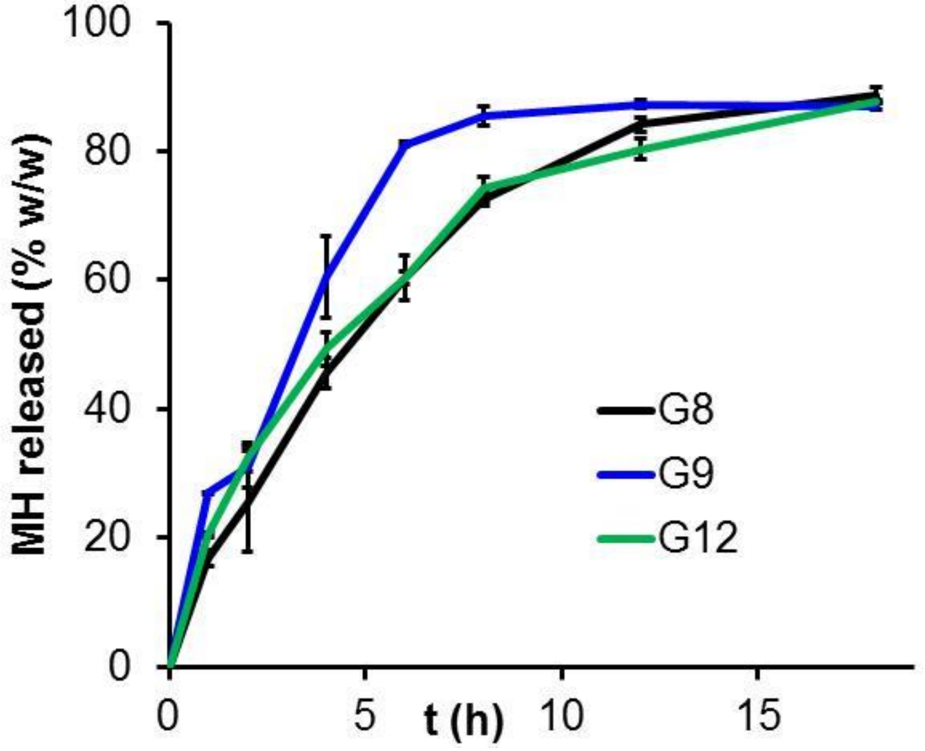

Figure S2. Metformin (MH) release profiles of formulations G8, G9 and G12 (n=3).

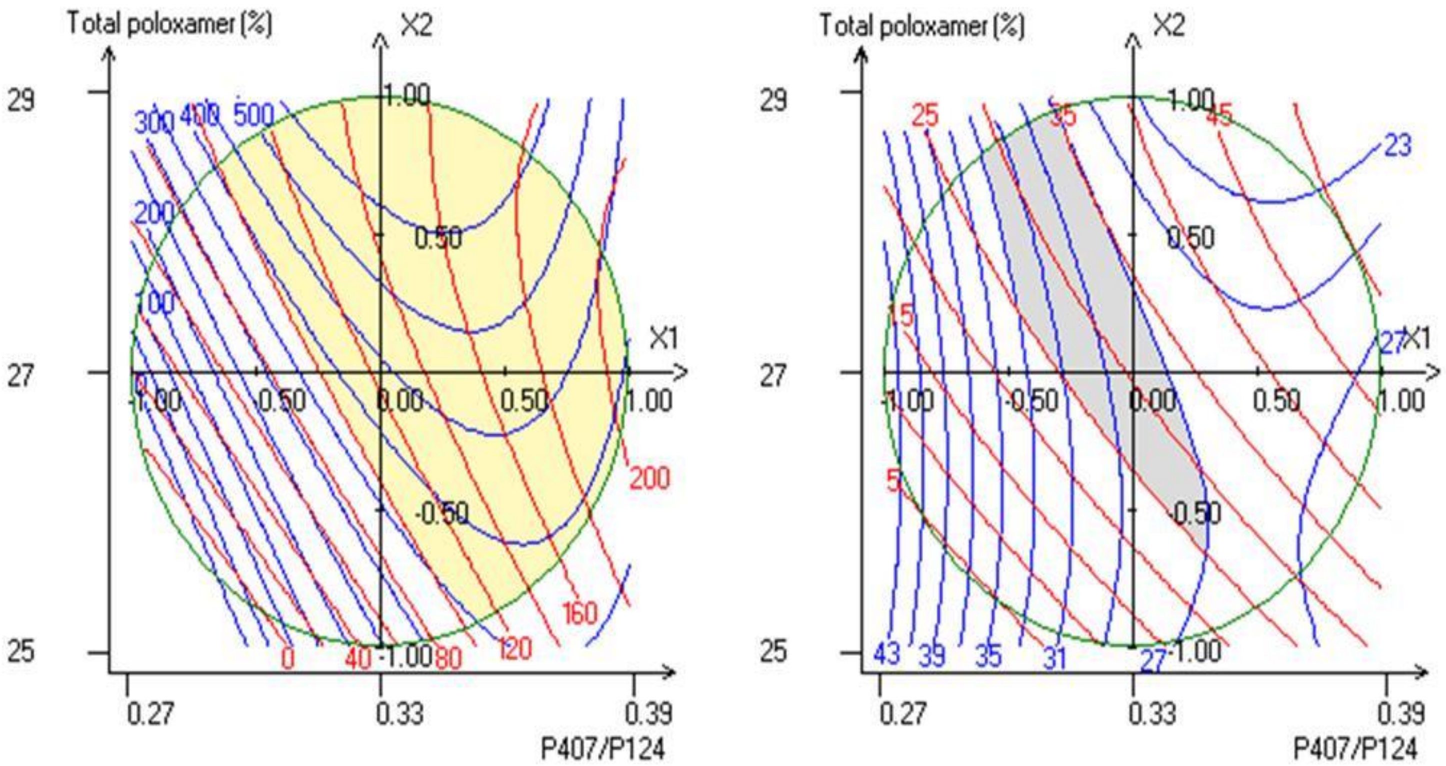

Figure S3. Contour plots of Y2-Y3 (left) (the yellow area corresponds to Y2>100 mPa s and Y3>350 Pa) and Y4-Y6 (right) (the grey area corresponds to 27<Tgel<33 °C and gelation rate >25 mPa).

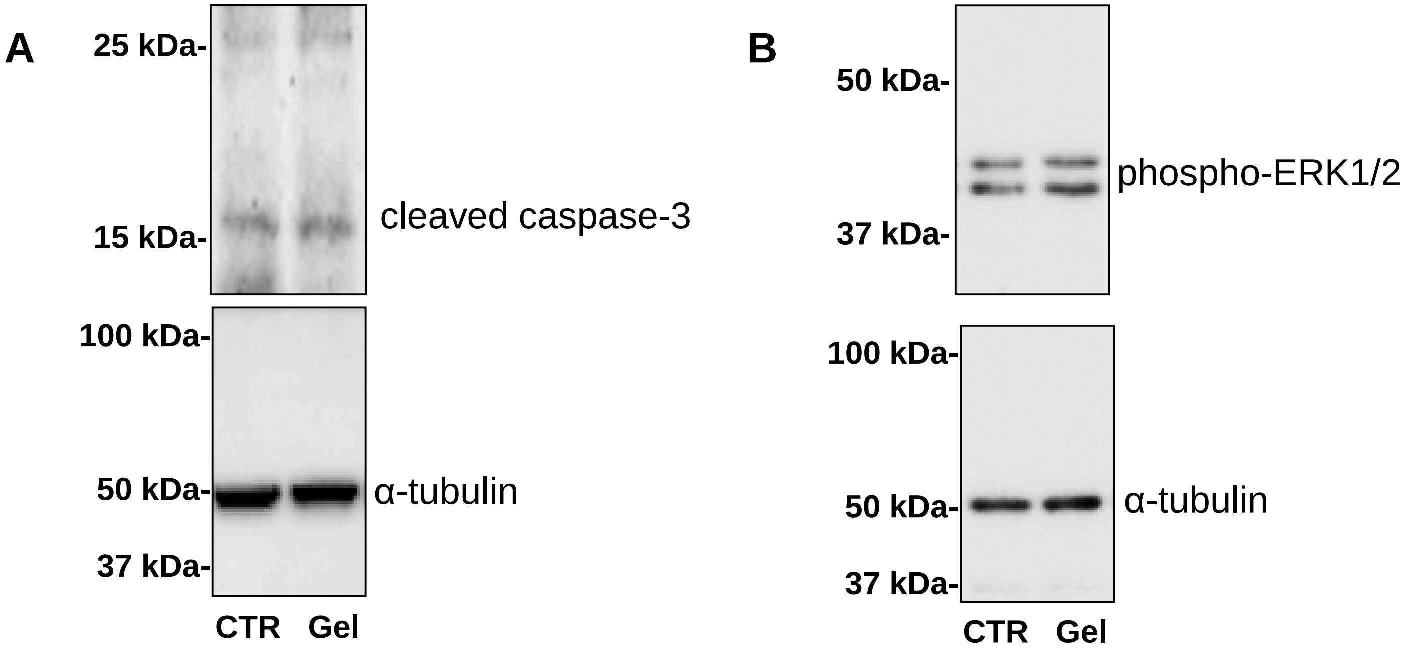

Figure S4.

A) Representative Western blot of extracts from MDA-MB-231 tumors developed by untreated mice (CTR) or mice treated with placebo (Gel), using the antibody against the active fragment of caspase-3 (cleaved caspase-3).

B) Representative Western blot of extracts from MDA-MB-231 tumors developed by untreated mice (CTR) or mice treated with placebo (Gel), using the antibody against phospho-ERK1/2.

Blots were stripped and reprobed with anti-*α*-tubulin antibody to normalize for differences in protein loading.
